# Associations of ADHD traits, sleep/circadian factors, depression and quality of life

**DOI:** 10.1136/bmjment-2025-301625

**Published:** 2025-07-14

**Authors:** Siddhi Nair, Neha Deshpande, Catherine Hill, Samuele Cortese, Eus J W Van Someren, Sarah Laxhmi Chellappa

**Affiliations:** 1 Centre for Innovation in Mental Health, School of Psychology, Faculty of Environmental and Life Sciences, University of Southampton, Southampton, UK; 2 Clinical and Experimental Sciences, University of Southampton Faculty of Medicine, Southampton, UK; 3 Hampshire and Isle of Wight Healthcare Foundation NHS Trust, Southampton, UK; 4 Hassenfeld Children's Hospital at NYU Langone, New York University Child Study Center, New York, New York, USA; 5 Department of Precision and Regenerative Medicine and Ionian Area, University of Bari "Aldo Moro", Bari, Italy; 6 Netherlands Institute for Neuroscience, Amsterdam, The Netherlands; 7 Department of Psychiatry, Amsterdam UMC, Amsterdam, The Netherlands; 8 Department of Integrative Neurophysiology, Center for Neurogenomics and Cognitive Research, Amsterdam Neuroscience, Vrije Universiteit Amsterdam, Amsterdam, The Netherlands

**Keywords:** Adult psychiatry, Depression & mood disorders, Sleep

## Abstract

**Background:**

Individuals with attention deficit hyperactivity disorder (ADHD) are at a higher risk of depression and lower quality of life (QoL); however, it is unclear whether disrupted sleep and circadian rhythms mediate this increased risk.

**Objectives:**

We investigated whether disruption of self-reported sleep and circadian factors mediate the associations of ADHD traits with depression symptom severity and QoL.

**Methods:**

1364 participants (mean: 51.86 (SD=0.37) years, 75% women) from a large-scale cross-sectional online survey (Netherlands Sleep Registry) completed a sociodemographic questionnaire, the Adult ADHD Rating Scale, Hospital Anxiety and Depression Scale, Satisfaction With Life Scale (SLS) and Cantril Ladder (CL) (QoL measures), Insomnia Severity Index, Pittsburgh Sleep Quality Index and Munich Chronotype Questionnaire.

**Findings:**

Higher ADHD traits were significantly associated with depression symptom severity (p=0.03), lower QoL (p<0.001), insomnia severity (p<0.001), lower sleep quality (p<0.001) and later chronotype (p=0.01). No sleep or circadian factor significantly mediated the association of the severity of symptoms of ADHD and depression (all p>0.1). Conversely, only insomnia severity significantly mediated the association of ADHD traits and QoL (SLS: standardised β=−0.10, 95% CI (−0.12 to –0.04); CL: standardised β=0.103, 95% CI (0.04 to 0.16)).

**Conclusion:**

ADHD traits were associated with lower QoL and it was partially mediated by insomnia severity. Future studies targeting insomnia complaints in this population may help mitigate their depression complaints and improve their QoL.

**Clinical implications:**

Our results may help current clinical guidelines that do not typically link sleep/circadian complaints to QoL in ADHD assessment.

**WHAT IS ALREADY KNOWN ON THIS TOPIC**

- Attention deficit hyperactivity disorder (ADHD) traits are often associated with depression risk and lower quality of life (QoL). Similarly, sleep and circadian rhythm disruption (SCRD) is entwined with depression and decreased psychological well-being. However, it is unclear whether SCRD mediates the association of ADHD traits with depression risk and lower QoL.

**WHAT THIS STUDY ADDS**

- We show that insomnia severity (but not self-reported sleep quality, chronotype or social jetlag) predicts the association of ADHD traits with lower QoL. This is the first study to show that specific sleep problems can explain psychological distress experienced by middle-aged and older adults with ADHD traits.

**HOW THIS STUDY MIGHT AFFECT RESEARCH, PRACTICE OR POLICY**

- Our study indicates that targeting insomnia complaints in individuals with higher ADHD traits may help improve their QoL. Our results are therefore critical in the development of effective, scalable preventative and therapeutic support systems to improve the QoL in individuals with ADHD traits.

## Background

 Attention deficit hyperactivity disorder (ADHD) is a common neurodevelopmental condition with a worldwide-pooled prevalence of 5–7%.^1 2^ ADHD is characterised by differences in attention, hyperactivity, impulsivity, or a combination of these traits.^3^ ADHD often co-occurs with mental disorders, particularly depression,^4^ whereby up to 80% of adults with ADHD (18–35 years) report at least one episode of depression throughout their lifespan compared with age-matched and sex-matched individuals with neurotypical development.^5^ A meta-analysis of 18 cross-sectional studies (1615 individuals with ADHD, 3128 individuals with neurotypical development) showed a significant association between ADHD clinical diagnosis and depression among young adults (OR: 4.66, 95% CI (2.92 to 7.45)).^6^ Similarly, quality of life (QoL) may be perceived as negatively affected in individuals with ADHD .^7^ A recent meta-analysis including 23 studies of children with ADHD (4–18 years) showed that both parent-reported (Hedges’ *g* −1.67, 95% CI (−2.57 to –0.78)) and child-reported (Hedges’ *g* −1.28, 95% CI (−2.01 to –0.56)) health-related QoL was lower compared with children with neurotypical development.^7^ Despite the high prevalence of ADHD and its impact on quality of life management, biological factors contributing to these associations remain to be fully established. Of note, most studies on ADHD have primarily focused on children and adolescents. Thus, it is largely unknown if middle-aged and older people with ADHD also experience a heightened likelihood of depression risk and low QoL.

At least 25% of adults with ADHD self-report sleep disorders, including delayed sleep–wake phase syndrome, restless legs syndrome, daytime sleepiness and insomnia.^8^ The latter is the most common sleep disorder affecting the general population, with estimates of 43–83% among ADHD.^9^ A meta-analysis from 16 cross-sectional studies (722 children with ADHD and 638 age-matched and sex-matched children with neurotypical development) indicates higher bedtime resistance (z=6.94), greater sleep onset difficulties (z=9.38), night awakenings (z=2.15) and daytime sleepiness (z=1.96) in children with ADHD.^10^ Similarly, a meta-analysis of 13 cross-sectional studies revealed that adults with ADHD had longer sleep onset latency (standardised mean difference, SMD=0.80, 95% CI (0.46 to 1.14)) and lowered sleep efficiency (SMD=−0.68, 95% CI (−1.03 to –0.34)), compared with individuals with neurotypical development.^11^ Sleep disruption/curtailment contribute to cognitive and behavioural differences associated with ADHD but also intersect with the underlying physiology and may be interrelated to its physiology underpinnings. Similarly, ADHD traits may be associated with circadian disturbances. Studies of circadian rhythms in adults with ADHD indicate that ~30% report late chronotype, delayed circadian phase (as indexed by dim light melatonin onset) and higher risk of delayed sleep–wake phase syndrome.^12^ Sleep and circadian rhythm disturbances (SCRD) are bidirectionally intertwined with almost every category of mental disorder in a transdiagnostic manner.^13^ However, the majority of these studies focus on individuals with neurotypical development. Thus, it is currently unknown whether SCRD mediates the increased risk of depression and lower QoL in individuals with ADHD traits.

## Objectives

Here, we investigated whether disruption of sleep (eg, insomnia and low self-reported sleep quality) and circadian factors (eg, chronotype and social jetlag) mediate the association of ADHD traits with increased depression symptom severity and lower QoL. We used data from the Netherlands Sleep Registry (NSR), a large-scale online survey assessment platform with over 10,000 participants aged 18 years and above.

## Methods

### Participants

Data included here are from the NSR, an online survey dataset with >10,000 participants (aged 18 and older) from the general population (www.slaapregister.nl). Participants were recruited via media, advertisements and flyers distributed at healthcare institutions and conventions. The participation of people was voluntary, and all data were recorded anonymously.^14^ The only inclusion criterion was an age of 18 years or older. Participants completed a variable number of randomly selected questionnaires (of 34 online questionnaires) at their convenience, resulting in a varying number and set of completed questionnaires between participants. The NSR study commenced in February 2010 and was completed in November 2013.

In the present study, of the 3571 participants who completed the ADHD questionnaire, 1364 also fully completed the questionnaire battery of interest, which included questionnaires about insomnia (n=2354), sleep quality (n=2348), chronotype (n=1913), social jetlag (n=1899), depression (n=2490) and QoL (n=3251) (table 1).

**Table 1:** Sociodemographic and study characteristics (n=1364).

Demographic variables	M (95% CI)
Sex	0.8 (0.74 to 0.78)
Age	51.9 (51.13 to 52.59)
Marital status	2.2 (2.09 to 2.22)
Educational level	6.6 (6.50 to 6.65)
Employment	3.7 (3.55 to 3.83)
Adult ADHD Self-Report Scale	1.7 (1.59 to 1.75)
Hospital Anxiety and Depression Scale	9.1 (9.00 to 9.17)
Satisfaction with Life Scale	23.6 (23.25 to 24.0)
Insomnia Severity Index	9.4 (8.99 to 9.74)
Pittsburg Sleep Quality Index	7.2 (6.96 to 7.44)
Munich Chronotype Questionnaire	3.9 (3.80 to 3.92)

ADHD, attention deficit hyperactivity disorder.

### Measurements

#### Attention deficit hyperactivity disorder (ADHD)

ADHD traits were measured using the Adult ADHD Self-Report Scale. This tool was designed to help screen adults (aged 18 years and older) and is validated with the full version of the ASRS (an 18-item measure corresponding to the ADHD symptoms in the Diagnostic and Statistical Manuel, DSM-IV).^15^ The current measure consists of six items (four on inattention and two on hyperactivity), and participants were required to rate their core characteristics on a five-point Likert scale (0=never; 1=rarely; 2=sometimes; 3=often; 4=very often). The participants were classified based on their scores into no trait group (ASRS symptom score=0), moderate trait group (ASRS symptom score=1–3) and high trait group (ASRS symptom score≥4). The ASRS has moderate sensitivity (68.7%), high specificity (99.5%) and high total classification accuracy (97.9%). The cut-off score for potential ADHD traits were 4 and above.

#### Depression

Depression symptom severity was assessed using the Hospital Anxiety and Depression Scale (HADS). This 14-item self-reported scale includes 7 items for anxiety and 7 for depression with responses scored on a 4-point Likert scale, from 0 to 3, with higher scores representing worse symptoms. The maximum score attainable for each subscale is 21, with the cut-off score for the presence of the condition at 8. A score of 0–7 is in the normal range, 8–10 is mild to moderate and 11 ≥ is severe.

#### QoL measures

The Satisfaction with Life Scale (SLS) measures psychological well-being and is a short 5-item questionnaire aiming to measure cognitive judgements of an individual’s life satisfaction and does not measure affect (either positive or negative). The maximum score attainable is 35, and the higher the score, the better the individual’s life satisfaction and well-being.

The Cantril’s Self-Anchoring Ladder of Life Satisfaction (Cantril Ladder (CL)) measures life satisfaction by visualising an adult’s life in the best possible manner and explaining dreams and hopes for the future. This is followed by asking the opposite; for example, explaining and describing their worst fears and envisioning the worst-case scenarios. A score of four or below indicates ‘suffering’ and scoring more than seven equates to ‘thriving’. The higher the score, the better the individual’s life satisfaction and well-being.

#### Insomnia

The Insomnia Severity Index (ISI) is a 7-item questionnaire where participants choose options on a 5-point Likert scale ranging from 0 (not at all) to 4 (very severe/very much). The total score ranges between 0–28 with the cut-off score for likely insomnia being at least 10, and above 15 suggesting high risk of clinical insomnia indicating severe sleep disturbance and associated daytime symptoms.

#### Sleep quality

The Pittsburgh Sleep Quality Index (PSQI) assessed difficulties initiating and maintaining sleep. This 19-item self-report questionnaire includes 7 subscales: sleep latency, sleep duration, subjective sleep quality, habitual sleep efficiency, sleep disturbances, daytime dysfunction and use of sleeping medication. The questionnaire comprises both Likert-type and open-ended questions, and participants must answer how often over the past month they experienced sleep difficulties and rate their overall sleep quality. Scores for each question range from 0 (no difficulty) to 3 (severe), with the cut-off score of 5, and the values above the cut-off to indicate severe sleep difficulties. The maximum score possible on the questionnaire is 21. We assessed sleep-related measures (self-reported sleep quality, ISI) using well-validated questionnaires that have been used in adults with ADHD.^16^ Thus, the validity/reliability of sleep-related scales (PSQI, ISI) has been tested for adults with ADHD.

#### Chronotype and social jetlag

The Munich Chronotype Questionnaire (MCTQ) is a self-rated scale that assesses an individual’s entrainment of working and work-free days. It consists of 17 items, divided into 4 categories (work schedule, workday sleep schedule, free day sleep schedule and chronotype), regarding sleep–wake behaviour of individuals, attempting to distinguish weekday and weekend schedules. The MCTQ was designed to assess chronotype by evaluating the corrected midsleep on free days, which is the midpoint between sleep onset and offset on free days. Social jetlag was calculated using the difference between the midpoint of sleep on workdays and the midpoint of sleep on free days.

### Data analysis

Data were analysed using IBM Statistics V.29 and SAS V.9.4. Outliers (ie, values over three SD of the mean) were removed from the analyses. To standardise the dataset, all of the data were z-transformed. The data did not present with multicollinearity, and neither did it have independent errors. First, means and SD were calculated for the sociodemographic data (sex, age, marital status, education level and employment status) and the data obtained from the ASRS, ISI, PQSI, MCTQ, HADS, SLS and CL questionnaires. Additionally, percentages were also calculated for each category of the sociodemographic data. Next, we used Spearman correlations to assess the associations of ADHD traits, depression symptom severity, QoL, insomnia severity, self-reported sleep quality, chronotype and social jetlag. Linear and hierarchical regression models were performed to determine the effect of ADHD traits on depression symptom severity and QoL, while accounting for sociodemographic factors, insomnia severity, self-reported sleep quality, chronotype and social jetlag. Hierarchical multiple linear regression was carried out to identify the effects of ADHD traits on depression symptom severity and QoL, controlling for covariates of interest (model 1: sociodemographic variables age, sex, marital status, educational level, employment; model 2: sociodemographic variables and sleep/circadian predictors self-reported sleep quality, chronotype, social jetlag and insomnia severity).

The effect sizes for each were determined by the R^2^ values with 0.02, 0.13 and 0.26 indicating small, medium and large effect sizes, respectively. We assessed potential multicollinearity in the regression models by using methods such as correlation coefficients or variance inflation factor. If multicollinearity was present, we applied Ridge regression, which is a technique that adds a penalty to the regression coefficients and helps to reduce the impact of multicollinearity when using different instruments.^17^ We applied structural equation modelling, which involves two or more endogenous (observed) variables in the multivariate regression models. We used this approach as it allowed us to evaluate the relationships among multiple predictors and outcomes simultaneously. Finally, a mediation analysis was done using PROCESS (V.4, model 6: Hayes 2022) to identify whether sleep/circadian factors (ie, self-reported sleep quality, insomnia severity, chronotype and social jetlag) mediated depression symptom severity and QoL among individuals with ADHD traits. Two mediation analysis models were constructed: the first model included the sleep/circadian predictors. In the next step, we added the covariates sex, age, marital status, education level and employment status to create the second model. Of note, the addition of the covariates was justified by a significant increase in model fit relative to the loss of parsimony (using the Akaike information criterion). Our mediation analyses aimed to uncover potential mechanisms/pathways that explain the effect of ADHD traits influences depression symptom severity and QoL. A moderation analysis, on the other hand, would have identified moderators that change the strength or direction of the association of ADHD traits on depression symptom severity and on QoL outcomes. Both mediation and moderation analyses help dissect the relationships between variables, but they serve distinct functions. Mediation explains the pathway through which an effect occurs, answering the ‘how’ and ‘why’ of the targeted association, while moderation analysis examines when this association changes, highlighting the conditions that affect the strength of these relationships.^18^ Moreover, we used traditional mediation analyses instead of causal mediation analyses (eg, chained (serial) and parallel mediation models). The former provides the same effect estimates as causal mediation analysis, for example, single mediator models with a continuous mediator and a continuous outcome, as we did not use binary or time-to-event outcomes,^19^ which are best assessed with causal mediation analyses.

## Findings

### Correlational analysis

Higher ADHD traits were significantly associated with higher depression symptom severity (r=0.11, p=0.03) and lower QoL (SLS: r=−0.24, p<0.001; CL: r=−0.24, p<0.001) (online supplemental figure 1). Likewise, higher ADHD traits were significantly associated with higher insomnia severity (r=0.27, p<0.001), lower sleep quality (r=0.24, p<0.001) and later chronotyoe (r=0.08, p=0.01) (online supplemental figure 2), but not social jetlag (r=0.03, p=0.08).

### Hierarchical regression models

#### Depression

None of the sociodemographic predictors significantly contributed to depression severity (model 1: R^2^=0.07; all p>0.1; model 2: R2=0.07; all p>0.1). Likewise, none of the sleep/circadian predictors (model 2: R^2^=0.07; all p>0.09) nor ADHD traits (model 2: R^2^=0.07; p=0.11) significantly contributed to depression severity (table 2).

**Table 2:** Hierarchical regression results for depression symptom severity.

Variable	95% CI				
	B	SE	β	P value	R^2^
Model 1					0.07
Constant	−0.15	0.17			
Sex	−0.02	0.06	−0.01	0.71	
Age	0.002	0.002	0.03	0.33	
Marital status	−0.005	0.02	−0.01	0.79	
Education level	0.02	0.02	0.03	0.23	
Employment	−0.01	0.01	−0.04	0.11	
Model 2					0.07
Constant	−0.12	0.18			
Sex	−0.03	0.06	−0.01	0.61	
Age	0.001	0.002	0.002	0.44	
Marital status	−0.01	0.02	−0.01	0.77	
Education level	0.02	0.02	0.03	0.25	
Employment	−0.01	0.01	−0.04	0.10	
Insomnia Severity Index	0.04	0.05	0.04	0.40	
Pittsburgh Sleep Quality Index	0.08	0.05	0.08	0.09	
Munich Chronotype Questionnaire	−0.04	0.02	−0.04	0.09	
Social jetlag	0.00	0.01	0.00	0.48	
Adult ADHD Self-Report Scale	−0.04	0.03	−0.04	0.11	

Note: n=1364.

ADHD, attention deficit hyperactivity disorder.

#### Quality of life (QoL)

##### Satisfaction with Life Scale (SLS)

None of the sociodemographic predictors significantly contributed to SLS QoL (model 1: R^2^=0.35; p>0.09). However, insomnia severity and ADHD traits were significant contributors to SLS QoL (model 2: R^2^=0.35; respectively, p<0.001 and p=0.03). Sociodemographic factors alone had a medium effect (26% of the variance; model 1). In contrast, sleep/circadian predictors, sociodemographic factors and ADHD traits combined had a large effect (35% of the variance; model 2) (table 3).

**Table 3:** Hierarchical regression results for the Satisfaction with Life Scale.

Variable	95% CI				
	B	SE	β	P value	R^2^
Model 1					0.16
Constant	0.05	0.16			
Sex	0.19	0.05	0.08	<0.001	
Age	−0.002	0.002	−0.03	0.20	
Marital status	−0.17	0.02	−0.21	<0.001	
Educational level	0.04	0.02	0.06	0.06	
Employment status	−0.03	0.01	−0.09	0.07	
Model 2					0.35
Constant	0.02	0.17			
Sex	0.16	0.07	0.07	0.026	
Age	0.01	0.04	0.01	0.72	
Marital status	−0.15	0.02	−0.20	0.09	
Educational level	0.03	0.02	0.04	0.15	
Employment status	−0.03	0.01	−0.08	0.07	
Insomnia Severity Index	−0.19	0.06	−0.19	<0.001	
Pittsburgh Sleep Quality Index	−0.05	0.06	−0.05	0.38	
Munich Chronotype Questionnaire	−0.02	0.03	−0.02	0.51	
Social jetlag	0.00	0.01	0.01	0.59	
Hospital Anxiety and Depression Scale	−0.06	0.03	−0.05	0.08	
Adult ADHD Self-Report Scale	−0.07	0.03	−0.07	0.03	

Note: n=1364.

ADHD, attention deficit hyperactivity disorder.

##### Cantril Ladder (CL)

The sociodemographic predictors sex, age, marital status and educational level significantly contributed to CL QoL (model 1: R^2^=0.27; all p<0.02). Moreover, insomnia severity, depression symptom severity and ADHD traits significantly contributed to CL QoL (respectively, p=0.002, p=0.03 and p=0.03). Sociodemographic factors alone had a medium effect (16% of the variance; model 1). In contrast, sleep/circadian predictors, sociodemographic factors and ADHD traits combined had a large effect (27% of the variance; model 2) (table 4).

**Table 4:** Hierarchical regression results for the Cantril Ladder Questionnaire.

Variable	95% CI				
	B	SE	β	P value	R^2^
Model 1					0.16
Constant	1.45	0.08			
Sex	−0.08	0.03	−0.08	0.02	
Age	−0.05	0.02	−0.10	0.004	
Marital status	0.03	0.01	0.09	0.004	
Education level	−0.02	0.01	−0.08	0.015	
Employment status	0.00	0.01	0.03	0.41	
Model 2					0.27
Constant	1.45	0.08			
Sex	−0.07	0.03	−0.07	0.03	
Age	−0.04	0.02	−0.09	0.009	
Marital status	0.03	0.01	0.09	0.003	
Education level	−0.02	0.01	−0.08	0.01	
Employment	0.00	0.01	0.03	0.39	
Insomnia Severity Index	0.08	0.03	0.19	0.002	
Pittsburgh Sleep Quality Index	0.01	0.03	0.02	0.71	
Munich Chronotype Questionnaire	0.01	0.01	0.01	0.67	
Social jetlag	0.00	0.01	0.00	0.68	
Hospital Anxiety and Depression Scale	0.03	0.01	0.07	0.03	
Adult ADHD Self-Report Scale	0.03	0.02	0.08	0.03	

Note: N=1364.

ADHD, attention deficit hyperactivity disorder.

### Mediational analysis

#### Depression

Only insomnia severity significantly mediated the association of ADHD traits with depression severity (model 1: standardised β=0.05, bootstrapped 95% CI (0.01 to 0.06)). However, after adding the sociodemographic predictors, none of the sleep/circadian predictors (self-reported sleep quality, insomnia severity, chronotype and social jetlag) significantly mediated the association of ADHD traits with depression severity (model 2: standardised β=0.049, bootstrapped 95% CI (−0.03 to 0.07)).

#### Quality of life (QoL)

##### Satisfaction with Life Scale (SLS)

Chronotype and insomnia severity significantly mediated the association of ADHD traits with SLS QoL (model 1: standardised β=−0.11, bootstrapped 95% CI (−0.13 to –0.09)). However, after adding the sociodemographic factors, only insomnia severity significantly mediated the association of ADHD traits with SLS QoL (model 2: standardised β=−0.10, bootstrapped 95% CI (−0.13 to –0.08)).

##### Cantril Ladder (CL)

Only insomnia severity significantly mediated the association of ADHD traits with CL QoL before and after including the sociodemographic factors (standardised β=0.103, bootstrapped 95% CI (0.08 to 0.13)).

## Discussion

We showed that ADHD traits were associated with higher depression symptom severity, lower QoL, insomnia severity, lower self-reported sleep quality and later chronotype. Importantly, insomnia severity mediated the association of ADHD traits with QoL.

Our study focused on the associations of sleep/circadian factors in depression and QoL in middle-aged and older adults with ADHD traits, which is a largely under-researched theme. Currently, the majority of ADHD studies, including those assessing sleep and circadian health, are primarily focused on children and adolescents. In a nationally representative sample of US adults in 2023, an estimated 15.5 million (6%) had an ADHD clinical diagnosis and half of those received their diagnosis during adulthood.^20^ Only half of the adults with ADHD were receiving any ADHD intervention support, and only 10% of adults with ADHD were receiving support for co-occurring mental health problems. This context highlights the need for more research in adults with ADHD, as well as a better understanding of the sleep, circadian and mental health problems that can affect their QoL.

Approximately 30–35% of adults with ADHD experience at least one major depressive disorder episode throughout their lifespan, which is 15% higher than the risk observed in the general population.^21^ In a recent two-sample network Mendelian randomisation analysis that aimed to identify mental disorders causally related to ADHD, the genetic likelihood of ADHD was associated with an increased risk of major depressive disorder.^22^ Similarly, meta-analysis (ADHD: n=550 748; no ADHD n=14 546 814) yielded pooled ORs of 4.5 for major depressive disorder, suggesting a high co-occurrence of ADHD with major depression compared with non-ADHD.^23^ Similarly, a combined longitudinal study (n=8310) with two-sample Mendelian randomisation analyses indicated childhood ADHD was associated with a higher risk of depression in adulthood, due to a causal effect of ADHD genetic likelihood on major depression.^24^ Currently, there are no meta-analyses on adult ADHD and QoL. In a systematic review examining the association of adult ADHD and QoL in six studies, ADHD clinical diagnosis was consistently associated with an overall lower QoL.^25^ Similarly, in a multicentre study with young adults with ADHD and age-matched and sex-matched individuals with neurotypical development, ADHD clinical diagnosis was associated with lower QoL.^26^ Collectively, these findings suggest that ADHD is associated with an increased risk of depression and lower QoL in adulthood. Here, we show similar associations of ADHD traits with depression symptom severity and lower QoL in the general population. Our findings thus help identify the importance of mental health and QoL in individuals with ADHD traits, irrespective of a pre-existing ADHD diagnosis. Individuals with ADHD are diagnosed with sleep disorders at a rate eight times higher than the general population,^27^ manifesting in various ways such as delayed sleep onset, increased movement during sleep, daytime sleepiness and shorter sleep duration.^8^ A recent Swedish nationwide register-based study assessed sleep disorders, including insomnia, across five different age groups of ADHD and individuals with neurotypical development.^27^ Accordingly, 7.5% of individuals with ADHD (n=145 490, 2.25% of the cohort) had a sleep disorder and 47.5% had been prescribed sleep medication. Irrespective of age, individuals with ADHD had an increased risk of having a sleep disorder diagnosis (OR range=6.4–16.1) and a sleep medication prescription, particularly for insomnia (OR range=12.0–129.4) compared with individuals with neurotypical development.^27^ The circadian system may contribute to sleep disturbances, as up to a third of individuals with ADHD exhibit a later chronotype and delayed sleep–wake phase disorder,^28^ with evidence for disrupted daily rhythms.^29^ Here, we showed that ADHD traits were associated, to a lesser extent, with later chronotype, possibly because the latter often occurs in individuals with ADHD who experience delayed sleep–wake phase disorder.^12^ As the current study did not have information on delayed sleep–wake phase syndrome, this could explain the (relatively) weak association. For study feasibility, participants were not specifically instructed not to use alarm clocks, which could influence the reliability of chronotype assessment. Of note, social jetlag did not significantly mediate the association of ADHD traits and QoL.

Importantly, we show that adult ADHD traits and insomnia severity were significant predictors of lower QoL and that insomnia severity mediated this association. However, we did not observe such findings for depression symptom severity. This difference could be due to the older age group in our study, which often reports lower depressive symptoms compared with younger age groups^30^ and/or the absence of clinically diagnosed depression compared with a previous study.^30^ To our knowledge, there are no previous reports of the association and mediation of sleep/circadian factors in depression and QoL in ADHD. In one of the very few studies tangentially addressing this association in children and adolescents with ADHD (n=373, 10–19 years), self-reported sleep quality was lower in individuals with ADHD with mental health conditions compared with those without.^31^ Similarly, in a large cross-sectional study (n=4618, aged 18–64), mental and physical functioning, work productivity and healthcare use were adversely affected if ADHD clinical diagnosis and insomnia symptoms co-occurred.^32^ These findings combined suggest that insomnia severity may help explain the lower QoL in individuals with ADHD traits.

Low sleep quality and self-reported insomnia occur in up to 60% of individuals with ADHD, which is 2.5-fold more than that for individuals with neurotypical development.^33^ The physiology of SCRD in ADHD is complex and with limited understanding. Recent mechanistic insights suggest that insomnia risk genes are enriched in ADHD.^34^ SCRD is a risk factor for the subsequent development of mental disorders, such as depression, is one of the earliest signs of relapse and occurs both during and between acute episodes of illness, where sleep-circadian disruption and mental disorders co-vary.^13 35^ Of note, the reported insomnia severity effects could be related to other potential sleep-related confounders (eg, sleep apnea, periodic limb movement disorder, sleep medication use). Future studies are required to determine whether insomnia severity alone or combined with other sleep problems mediates the association of ADHD traits with QoL.

Potential explanations for our findings include differences in cognitive processing among individuals with ADHD, the role of sleep and circadian-related genes in ADHD, and age-related neurodegenerative changes. Adults with ADHD often experience differences in cognitive processing, such as attention, emotional processing and/or impulsivity, which may affect an individual’s beliefs, emotions and self-esteem.^36^ Adults with ADHD may thus experience a heightened likelihood of experiencing low sleep quality, insomnia complaints and low mood levels, all of which lead to reduced life satisfaction. This inter-relationship may depend on, for example, age and sex, such that adolescents and young adults and females may experience a greater risk of insomnia complaints, depression and low QoL.^13^ However, the complex interrelationship of sociodemographic, sleep, mental health and QoL factors remains to be fully established in individuals with ADHD. Although a comprehensive framework integrating sleep and circadian mechanisms with QoL in ADHD remains underdeveloped, growing evidence indicates their critical role. Recent evidence from a genetic association study with Mendelian randomisation indicates that ADHD was associated with a 2.5-fold reduction in sleep duration compared with individuals with neurotypical development.^34^ Furthermore, later timing of the most active (wrist-worn assessed) 10-hour period was associated with a twofold higher odds of ADHD, suggesting later circadian phase and delayed sleep timing.^34^ Such mechanistic pathways may underpin the effects of, for example, insomnia severity on QoL. At last, age-related neurodegenerative changes, including glaucoma, can commonly alter light perception in this population due to their impact on retinal ganglion cell (RGC) function, potentially leading to compromised sleep, circadian rhythms and mood. In a cross-sectional study with ~150 middle-aged and older adults with glaucoma, depression symptom severity was strongly associated with RGCs loss, increasing abruptly above a threshold of 15%, potentially due to clock-related gene polymorphisms.^37^ Such findings suggest that RGCs loss in glaucoma may affect non-visual photic transduction and lead to mood disturbances. However, the age distribution of our sample makes it unlikely that glaucoma will be present in a substantial number of participants.^38^

Limitations of this study include the age range (primarily middle-aged adults) and (mostly) white females, which limits the generalisability of the findings. Further studies with different age groups, racial/ethnic groups and more men are required to estimate the magnitude of our reported effects in other age and sex groups. We did not assess objective markers of sleep quality (eg, wristworn-assessed sleep parameters) and circadian phase (eg, dim light melatonin onset), known to affect depression symptom severity and QoL, and this may limit an in-depth understanding of depressive physiology and ADHD traits. Lastly, the cross-sectional design of this study does not allow for causal testing, and while the mediation analysis allows inference of the mediating role of, for example, insomnia on the association of ADHD traits with QoL, causality cannot be established. Future longitudinal studies are required to establish a potential causal role of insomnia in the association of ADHD and QoL.

In summary, our study shows that ADHD traits were associated with lower QoL, and this effect was partially mediated by insomnia severity. Future studies targeting insomnia complaints in individuals with ADHD traits may help mitigate their mental health complaints and improve QoL.

## Clinical implications

Given the critical link among ADHD traits, insomnia severity and mental disorders, future mechanistic studies including preclinical and human laboratory studies are needed to understand this complex interplay. Such understanding will be critical in the development of effective, scalable preventative and therapeutic support to improve the QoL in individuals with ADHD traits. Cognitive–behavioural therapy for insomnia (CBT-I) is highly effective in improving sleep quality, reducing sleep-onset latency, reducing depressive symptoms and enhancing overall well-being, and it is the currently recommended main non-pharmacological treatment for insomnia in adults.^39 40^ CBT-I in adults with ADHD can reduce insomnia severity following treatment, although such improvements may not persist at follow-up, which indicates the need for further research on long-term effectiveness.^41^ Sleep restriction therapy, a key single component of CBT, can cost-effectively reduce insomnia symptoms.^42^ Recent studies have combined CBT-I with circadian rhythm support, which enhanced effects on mood and might be particularly relevant to ADHD.^43 44^ However, randomised clinical trials are needed to determine their effectiveness in adults with insomnia symptoms and ADHD. A better understanding of how sleep and circadian factors affect mental health and QoL in adults with ADHD will ultimately help current clinical guidelines that do not link sleep/circadian complaints in ADHD assessment.

## Supplementary material

10.1136/bmjment-2025-301625 online supplemental file 1

## Data Availability

Data are available on reasonable request.
