# Supplementary material for: Associations of ADHD traits, sleep/circadian factors, depression and quality of life
Source: BMJ Ment Health. 2025 Jul 14;28(1):e301625. doi: 10.1136/bmjment-2025-301625 (PMC12273136; doi:10.1136/bmjment-2025-301625)
Supplement: online supplemental file 1 [file bmjment-28-1-s001.pdf]

Supplementary file for:

Associations of ADHD traits, sleep/circadian factors, depression, and quality of life

Siddhi Nair[1#], Neha Deshpande[1#], Catherine M. Hill[2], Samuele Cortese[1,3,4,5]*, Eus Van Someren[6##], Sarah Laxhmi Chellappa[1*##]

[1] Centre for Innovation in Mental Health, School of Psychology, Faculty of Environmental and Life Sciences, University of Southampton, Southampton, United Kingdom.

[2] School of Clinical and Experimental Sciences, Faculty of Medicine, University of Southampton, Southampton, UK; Department of Sleep Medicine, Southampton Children's Hospital, University Hospital Southampton NHS Foundation Trust, Southampton, United Kingdom.

[3] Solent NHS Trust, Southampton, United Kingdom.

[4] Hassenfeld Children's Hospital at NYU Langone, New York University Child Study Center, New York City, New York, United States.

[5] DiMePRe-J-Department of Precision and Regenerative Medicine-Jonic Area, University of Bari "Aldo Moro", Bari, Italy.

[6] Netherlands Institute for Neuroscience, Department of Sleep and Cognition, Amsterdam, the Netherlands; Departments of Integrative Neurophysiology and Psychiatry, Center for Neurogenomics and Cognitive Research, VU University, Amsterdam UMC, Amsterdam Neuroscience, Amsterdam, the Netherlands.

[#] Shared first authors

[##] Shared senior authors

[#] Correspondence: S.L.Chellappa@soton.ac.uk; e.j.w.someren@vu.nl

This file contains:

Supplementary figure 1

Supplementary figure 2

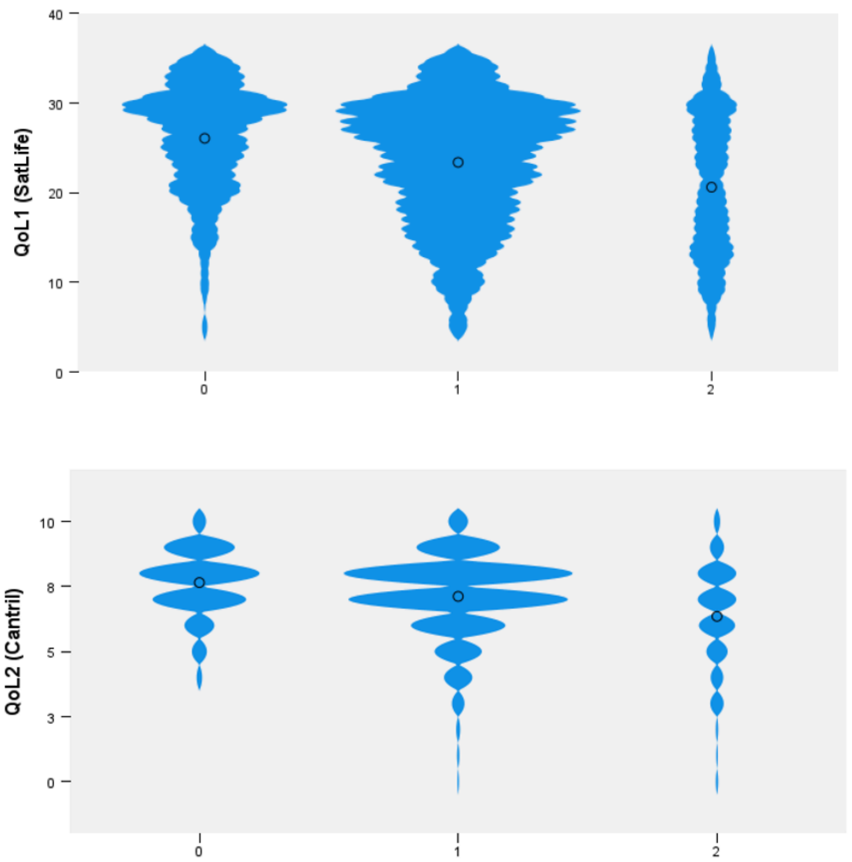

Supplementary Figure 1. Violin plots of the associations of ADHD traits with depression severity and quality of life. Higher ADHD traits were associated with higher depression symptom severity (Hospital Anxiety and Depression Scale [HADS], upper panel) and with lower quality of life (Satisfaction with Life scale [QoL1], middle panel; Cantril Ladder [QoL2], bottom panel). The x-axis indicates the Adult ADHD Self-Report Scale grouped based on the questionnaire's cutoff thresholds (0= traits, 1= moderate traits, 2= higher traits), and the y-axis indicates the corresponding depression and quality of life questionnaire scores. Note: for the upper panel, higher levels indicate higher depression symptom severity, and for the middle and bottom panels, higher levels indicate better quality of life.

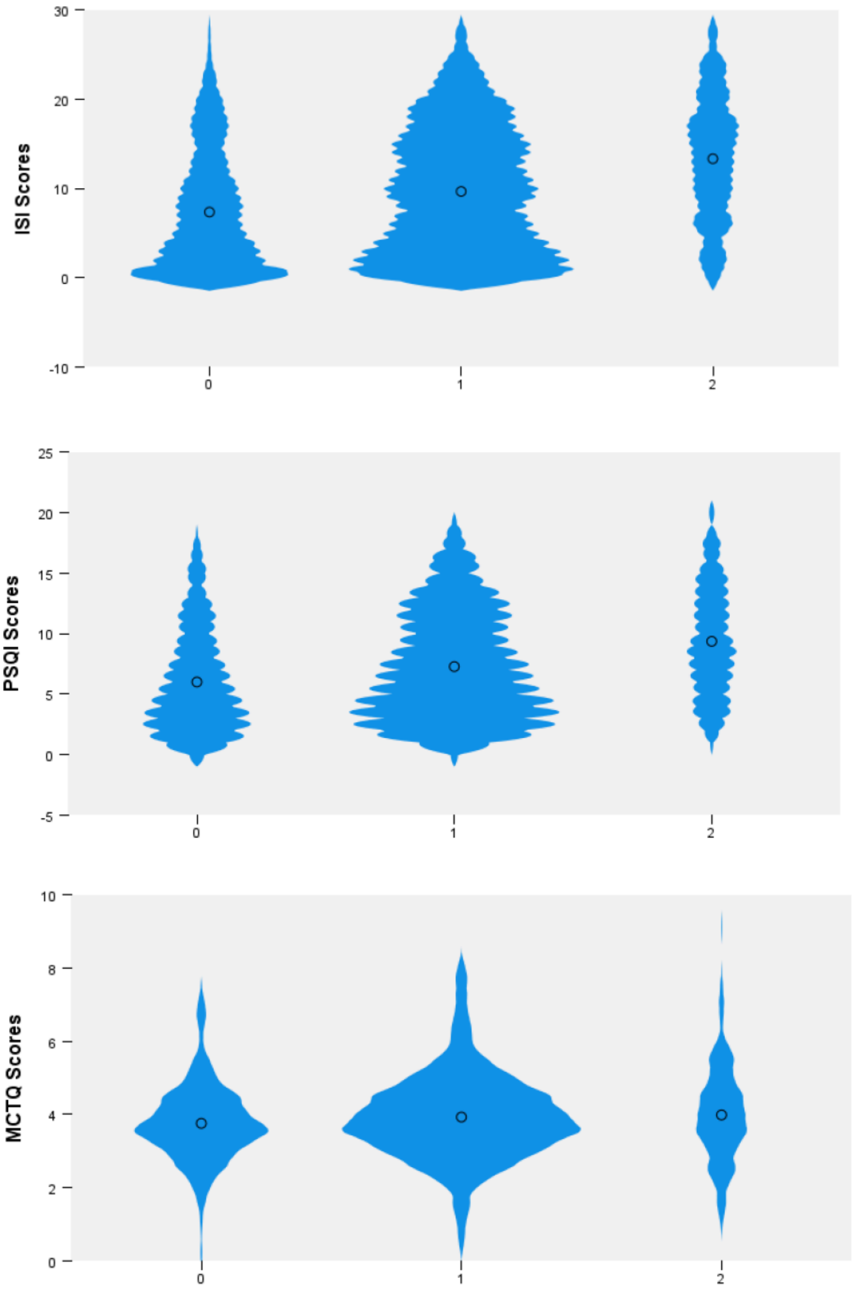

Supplementary Figure 2. Violin plots of the associations of ADHD traits with sleep and circadian factors. Higher ADHD traits were associated with higher insomnia severity (Insomnia Severity Index [ISI], upper panel), lower self-reported sleep quality (Pittsburg Sleep Quality Index [PSQI], middle panel), and later chronotype (Munich Chronotype Questionnaire [MCTQ], bottom panel). The x-axis indicates the Adult ADHD Self-Report Scale grouped based on the questionnaire's cutoff thresholds (0= no traits, 1= moderate traits, 2= higher traits), and the y-axis indicates the corresponding sleep and circadian questionnaires. Note: for the upper panel, higher levels indicate higher insomnia severity, for the middle panel, higher levels indicate worse self-reported sleep quality, and for the bottom panel, higher levels indicate later chronotype.
